# Inhalant Mediated Allergy: Immunobiology, Clinical Manifestations and Diagnosis

**DOI:** 10.1007/s12016-025-09053-2

**Published:** 2025-04-15

**Authors:** Ki Lam, Elaine Au, W. K. Ip, Jenna K. Tam, Patrick S. C. Leung

**Affiliations:** 1 Division of Clinical Immunology, Department of Pathology, Special Administrative Region, Queen Mary Hospital, Hong Kong, China; 2 Institute of Society and Genetics, University of California, Los Angeles, CA 90095 USA; 3 Division of Rheumatology/Allergy and Clinical Immunology, University of California, Davis, CA 95616 USA

**Keywords:** Allergy, Diagnosis, Asthma, Allergic rhinitis, Immunotherapy

## Abstract

Inhalant allergen–mediated respiratory diseases, including asthma and allergic rhinitis, have become increasing global health issues. While air pollution is believed to favor allergic sensitization and intensify clinical symptoms of allergy, allergen sensitization can vary highly with geographical location, climate, and lifestyle differences. Pollen sensitization is higher in European countries, while dust mite is more common in regions with high humidity. Domestic pet sensitization is on the rising trend in industrialized nations, but the paradoxical effect of intensive cat exposure in early childhood is also observed. Clinical management of inhalant allergic diseases has greatly benefited from the immunological and mechanistic understanding of pathophysiology. In this review, we discuss the current knowledge on inhalant mediated allergic disorders with emphasis on (1) the major immune cells and relevant chemokines and cytokines in the sensitization and effector phase with aeroallergen exposure, (2) their manifestation in asthma and allergic rhinitis, (3) characterization of inhalant allergens, (4) chemical contributions to the development of allergic diseases, and (5) clinical diagnosis of aeroallergen sensitization and management of inhalant allergy. Knowledge on the role of Th2 skewing, IgE, basophil, mast cells, and eosinophils in respiratory allergic diseases are fundamental in the diagnosis and management of these disorders. Skin test, basophil activation test, and specific IgE component–resolved diagnostics are used for diagnosis and facilitate further management. Advances in the development of biologics and allergen-specific immunotherapy will strategize the future approaches in the clinical care of respiratory allergic diseases.

## Introduction

Exposure to aeroallergens poses a significant burden to respiratory diseases. Sensitization to aeroallergens increases the risks of developing asthma and rhinitis. Globally, more than 260 million patients are diagnosed with asthma, accounting for a minimum of 359,000 deaths each year [1]. Allergic rhinitis accounts for up to 16–25% of the population in developed countries [2–4]. The prevalence increases rapidly in developing countries such as China (8.7 to 24.1% in eleven major cities) [5]. Increasing industrialization and air pollution further impact the pathophysiology and epidemiology of respiratory allergic diseases [6, 7]. Moreover, escalating global warming intensifies air pollution and prolongs pollen season [8–10], which can increase the risk of allergic sensitization and also exacerbate asthma and allergic rhinitis.

Understanding the allergen sensitization profiles in different localities is necessary for proper patient management [11–13]. Dust mite sensitization is more common in countries with high humidity (up to 65% in East China, Portugal) [2, 14, 15], while that for pollen is higher in other European countries (grass pollen allergy reaches 71%) [15]. With increasing domestic petting in industrialized nations [2, 15, 16], cat and dog sensitizations are becoming more prevalent. On the other hand, there are reports on paradoxical effects of intensive cat exposure in early childhood [16]. Studies have also demonstrated the significant involvement of Th2 skewing, IgE, basophil, mast cells, and eosinophils on the pathogenesis of respiratory allergic diseases [8, 17]. The modulation of regulatory T cells, regulatory B cells, and allergen-specific IgG4 is important for the management of atopic diseases [13, 18]. Diagnosis of allergen sensitization relies on skin tests and blood tests such as specific IgE and basophil activation tests [12, 13]. Altogether, these form the basis for allergen immunotherapy and biologics [13, 19, 20].

In this review, we discuss the role of allergic sensitization in respiratory allergic diseases, their clinical manifestation in allergic rhinitis and asthma, and common inhalant allergens and also present a comprehensive scope on the diagnosis of this growing health concern.

## Biological Responses upon Exposure to Inhalant Allergens

### IgE-Mediated Allergy

**Fig. 1:**
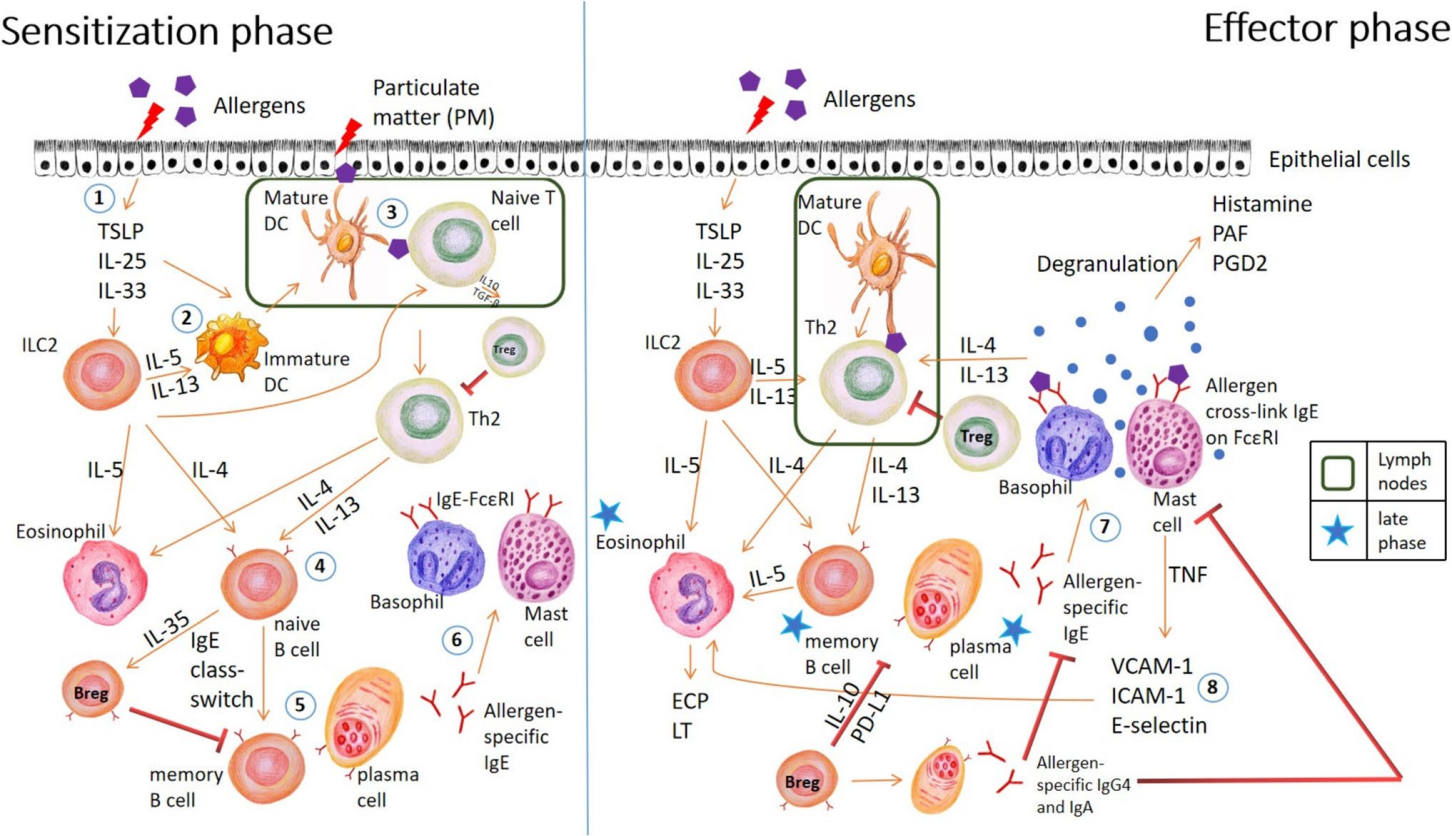
Cascade of immunopathological response in the sensitization and effector phase to inhalant allergens. Upon exposure to inhalant allergens, atopic individuals mount a sequence of immunological responses which can be schematically represented as follows: (1) Activated innate immune response in epithelial cells leads to release of cytokines such as TSLP, IL- 25, IL- 33, which activates tissue-resident ILC2 s to release IL- 5 and IL- 13. (2) Immature DCs are recruited to the site of inflammation and differentiate into proallergic type 2 DCs. (3) After internalization of the allergens, activated type 2 DCs migrate towards the draining lymph nodes and present antigen to naive CD4 + T cells in a MHC-II dependent manner, promoting their differentiation into Th2 cells. (4) IL- 4 and IL- 13 produced by Th2 stimulate IgE class switch in naive B cells. Naive B cells differentiate into IgE-producing plasmablasts and plasma cells. (5) Secreted allergen-specific IgE binds to FcεRI on the surface of mast cells and basophils, resulting in their sensitization. (6) Upon subsequent exposure, cross-linking of FcεRI-bound IgE on mast cells and basophils by allergen results in their activation and degranulation. Pro-inflammatory mediators are histamine, PAF, PGD2, and TNF. They interact with sensory nerves, blood vessels and glands in nasal and bronchial epithelium resulting in early-phase symptoms of allergic rhinitis and asthma. (7) Cytokines produced by mast cells (e.g., TNF) upregulate endothelial expression of leukocyte adhesion molecules, e.g., E-selectin, ICAM- 1, and VCAM- 1, further recruit eosinophils, basophils, T cells, and monocytes, and further enhance the allergic response at the site. This results in late-phase response. ILC2, group 2 innate lymphoid cells; DC, dendritic cells; MHC-II, major histocompatibility complex class II; FcεRI, high-affinity IgE receptors; PAF, platelet-activating factors; PGD2, prostaglandin D2; LT, leukotriene; TNF, tumor necrosis factors; ICAM- 1, intracellular adhesion molecules- 1; VCAM- 1, vascular cell adhesion molecules- 1; ECP, eosinophil cationic protein; Treg, regulatory T cells; Breg, regulatory B cells

Allergic rhinitis and asthma are diseases caused by inhalant allergens that triggered the generation of allergen specific IgE. Immunological responses to environmental allergens [8, 17, 21–26] are orchestrated with a prior sensitization phase, followed by the effector phase upon subsequent exposure to the same allergen. The overview of the mechanisms is illustrated in Fig. 1.

### Sensitization Phase

Extensive literature [6–9, 16, 17, 25–34] has documented that both intrinsic (including genetic predisposition, epigenetic modifications, human microbiome) and extrinsic factors (allergen type, dose, route and exposure duration) are necessary in triggering allergic sensitization [8, 17, 35]. Environmental factors such as exposure to tobacco smoke and microbial infections can alter DNA methylation [8, 28, 32] of genes that are associated with allergy and total serum IgE level. Furthermore, the human microbiome also exerts impact on allergic diseases, as corroborated by the “hygiene hypothesis” [8, 36]. Asthma exacerbations can be triggered by viral respiratory infections such as respiratory syncytial virus (RSV), rhinovirus, and human metapneumovirus [16]. Der p 1, the major allergen from dust mites, is an active cysteine protease that can disrupt tight junctions in epithelial cell layers [16, 27, 30]. Air pollutants such as diesel exhaust particulate matter can cause toxic irritation of the mucous membranes, subsequently disrupting epithelial integrity and thus allowing more antigens to enter the deeper mucosa [7–9, 16, 27, 29, 33]. The major cat allergen, Fel d 1, is airborne most of the time in homes with cats, so the quantity inhaled can be 100 times higher than in quantity than mite or pollen allergens [16, 30].

The nasal and skin epithelium are the first sites of allergen encounter and thus provide a critical physical barrier between the environment and host. Disruption of epithelial integrity by allergens activates downstream signaling cascades, like nuclear factor kappa-B (NF-kB) and mitogen-activated protein kinase (MAPK) pathways [8, 30]. Activated innate immune response in epithelial cells leads to the release of cytokines such as TSLP, IL- 25, IL- 33, and chemokines (like CCL2, CCL20). Besides the recruitment of immature dendritic cells (DCs) to the site of inflammation and promoting their differentiation into proallergic type 2 DCs, epithelial-derived mediators also activates tissue-resident group 2 innate lymphoid cells (ILC2 s) to release IL- 5 and IL- 13 [13, 37].

After the internalization of the allergens, activated type 2 DCs migrate towards the draining lymph nodes and present antigen to naive CD4 + T cells through major histocompatibility complex class II (MHC-II) [16, 21, 29, 30]. This promotes their differentiation into Th2 and T follicular helper (Tfh) cells [12, 22]. Th2 and Tfh cells produce Th2 cytokines like IL- 4 and IL- 13, which activate Janus-family tyrosine kinase (JAK; JAK 1 and JAK3) and lead to phosphorylation and activation of signal transducer and activator of transcription- 6 (STAT6) in naive B cells. This results in their class-switch to IgE in a CD40/CD40L interaction-dependent manner. They then differentiate into IgE-producing plasmablasts and plasma cells. Secreted allergen-specific IgE binds to high-affinity receptors (FcεRI) on the surface of mast cells and basophils, resulting in their sensitization.

Furthermore, IL- 4 and IL- 13 can induce the expression of transcription factor GATA binding protein 3 (GATA3) upon STAT6 activation in Th2 cells [12, 16, 21, 30]. STAT6 and GATA3 bind to the regulatory regions of Th2 cytokine locus and induce the gene expression of IL- 4, IL- 5, and IL- 13, resulting in positive feedback, a process known as Th2 polarization. Epithelial-derived mediators like TSLP also directly activate Th2 cells and increase the survival of naive CD4 + T cells and memory Th2 cells via phosphorylation of JAKs/STAT5 signaling.

Memory ILC2 s are generated from naïve ILC2 in the presence of IL- 33 and TSLP [37]. Memory ILC2 s are elevated in patients with asthma and atopic dermatitis [13, 37]. Despite being the first sensors of epithelial-derived mediators, ILC2 s do not express rearranged receptors and thus lack antigen recognition as Th2 cells (Table 1) [38]. Besides expressing receptors for TSLP, IL- 33, and IL- 25, ILC2 s also express common gamma chain (γc) family of cytokine receptors like IL- 2R and IL- 7R. ILC2 s proliferate upon exposure to allergens and produce IL- 5 and IL- 13 via the activation of the JAKs/STAT or MARK/NF-kB pathways. Naive ILC2 s express IL- 5 and IL- 13 mRNA but secrete very little cytokines and upregulate those mRNAs upon activation, whereas memory ILC2 s maintain elevated IL- 5 and IL- 13 mRNA and secrete cytokines when activated [37].

**Table 1:** Comparison between Th2 cells and innate lymphoid cells 2 (ILC2)

	Th2 cells	ILC2
Classification	Adaptive immune system	Innate immune system
Origin	CD3 + T cells (developed from common lymphoid progenitors)	Common lymphoid progenitors
Presence of rearranged receptors	Yes - Allow antigen recognition	No
Other cell surface receptors	CD3 IL- 4 receptor (IL- 4R) CCR3, CCR4 CRTH2	Common gamma chain (γc) family of cytokine receptors, e.g., IL- 2R and IL- 7R Receptors for TSLP, IL- 33, IL- 25
Activation	MHC II: peptide complex IL- 4	IL- 7, TSLP, IL- 33, IL- 25
Characteristic transcription factor	STAT6 GATA3	GATA3
Cytokines produced and functions	IL- 4, IL- 5, IL- 13 - Activate and recruit mast cells, basophil, eosinophil, ILC2 - Positive feedback for Th2 cells	IL- 5, IL- 13 - Activate and recruit mast cells, basophil, eosinophil, Th2 cells - Positive feedback for ILC2
Memory property	Yes	Yes

*CCR* chemokine receptors, *CRTH2* chemoattractant receptor-homologous molecule expressed on Th2 cells, *TSLP* thymic stromal lymphopoietin, *MHC* major histocompatibility complex, *STAT6* signal transducer and activator of transcription 6

In summary, allergen sensitization involves a complex cascade orchestrated by multiple immunological players that leads to the activation of antigen presenting cells and cytokine milieu that favor the production of allergen specific IgE generating plasmablasts, plasma cells, and memory B cells that are ready to take on when the allergen strikes again.

### Effector Phase

Upon subsequent exposure to the same allergen, cross-linking of FcεRI-bound IgE on mast cells and basophils by allergens leads to a highly orchestrated process of activation, degranulation, and release of proinflammatory mediators [8, 12, 19, 21]. Briefly, protein tyrosine kinases such as Fyn, Lyn, and Syk are involved. Lyn phosphorylates immunoreceptor tyrosine–based activation motifs (ITAMs) of FcεRI β and γ chains, thereby recruiting Syk to ITAMs of γ chains. Activated γ chains phosphorylate and activate adaptor molecules and enzymes of phospholipase Cγ (PLCγ). This increases phosphatidylinositol bisphosphate breakdown into inositol triphosphate (IP3) and diacylglycerol (DAG), generating calcium ion (Ca^2+^) and protein kinase C (PKC). PKC phosphorylates the myosin light chain of actin-myosin complexes beneath the plasma membrane of mast cells and basophils [23]. The granule membranes fuse with plasma membranes, resulting in degranulation. In mast cells, soluble *N*-ethylmaleimide–sensitive factor attachment protein receptor (SNARE) is important in the release of inflammatory mediators by mediating the fusion of secretory vesicles with the cell membrane. Mast cell degranulation is regulated by tomosyn- 1 (also known as STXBP5). During mast cell activation, phosphorylated tomosyn- 1 dissociated from syntaxin 4 (STX4), associated with STX3 with the presence of PKCδ [39], and inhibited mast cell degranulation.

The recruitment of basophils to their extravascular target sites requires the adhesion to the vascular endothelium, followed by trans-endothelial migration [40]. CCL2, CCL5, and CCL11 produced by the endothelial cells are necessary for attracting basophils to inflammatory sites. CCL11 (eotaxin) binds to the chemokine receptor CCR3 on basophils, mast cells, and eosinophils. CCL5 (RANTES) binds with a higher affinity to chemokine receptors CCR1, which is present on both basophils and eosinophils, than CCR3. CCL2 binds to chemokine receptor CCR2 that is present on basophils only [40]. In mast cells, the deposition of nucleotide-binding oligomerization domain 3 (NLRP3) and apoptosis-associated speck-like protein containing a CARD (ASC) forms a protein complex, granulosome [41]. It chaperones the granules to the cell surface. The coordination between phosphatases, kinases, and adaptor proteins induces the allergic early-phase response [42].

#### Early-Phase Allergic Response

Typically, early-phase response occurs within minutes upon allergen exposure and lasts for 1 to 2 h in allergic individuals [12, 13, 17, 27]. Degranulation of mast cells and basophils releases a lot of pro-inflammatory mediators: preformed mediators like histamine and tryptase; lipid mediators like prostaglandin D2 (PGD2), leukotriene (LT) C4, D4, E4, and platelet-activating factors (PAF); cytokines like tumor necrosis factors (TNFs), IL- 4, IL- 5, and IL- 13. They interact with sensory nerves, blood vessels, and glands in nasal and bronchial epithelium [8, 17, 21], resulting in early-phase symptoms of allergic rhinitis and asthma. Furthermore, they can further damage the epithelial integrity of the epithelial mucosa, further intensifying allergic response cascade. IL- 4 and IL- 13 also further stimulate Th2 cells.

#### Late-Phase Allergic Response

Late-phase response typically sets in from 2 to 4 h and usually peaks by 24 h [12, 21]. A late phase reaction is due to the inflammatory activity of recruited and activated inflammatory cells and continuous mediator production by mast cells [5]. Cytokines produced by mast cells (e.g., TNF) upregulate the endothelial expression of leukocyte adhesion molecules like E-selectin, intracellular adhesion molecules- 1 (ICAM- 1), vascular cell adhesion molecules- 1 (VCAM- 1), recruiting eosinophils, basophils, T cells, and monocytes. IL- 5 produced by Th2 cells increases the survival of eosinophils, while IL- 9 drives mast cell differentiation and survival. Chemokines secreted by epithelial cells, including CCL5, CCL11, thymus, and activation-regulated chemokine (CCL17), also promote the recruitment of eosinophils, basophils, and Th2 cells [8, 21, 22].

Eosinophils play an important role in late-phase response [13, 19]. Upon activation, they degranulate and release preformed eosinophil peroxidase, eosinophil collagenases, major basic protein (MBP), and eosinophil cationic protein (ECP); LT C4, D4, and E4; PAF; and IL- 3, IL- 4, IL- 5, IL- 13, and transforming growth factors (TGF) α and β [43]. Eosinophil peroxidase and MBP trigger the histamine release from mast cells, while eosinophil collagenases remodel connective tissues. ECP is a neurotoxin, whose level correlates with severity of allergic disease. IL- 5 promotes the activation and survival of eosinophil in an autocrine matter. IL- 3 and IL- 13 further stimulate eosinophil production by bone marrow, while TGF α and β increase epithelial proliferation. Overall, the symptoms manifest as a continuum of early-phase response.

Repetitive allergen exposure causes persistent activation of allergen-specific Th2 cells by dendritic cells and other antigen-presenting cells [17]. The large amount of Th2 cytokines produced by activated Th2 cells result in enhanced and sustained activation of mast cells and basophils, with increased release of their mediators. This is known as “priming,” with which progressive lower amounts of the allergen are needed to elicit symptoms [17] and can result in chronic allergic inflammation. For example, patients with allergic rhinitis become symptomatic with exposure of exceptionally low pollen levels towards the end of pollen season.

## Inhalant-Mediated Allergic Diseases

Inhalant allergens cause activation of lymphocytes, basophils, mast cells, and eosinophils, and thus their release of mediators and cytokines. They orchestrate cascades of clinical manifestations in atopic conditions like allergic rhinitis and asthma.

### Allergic Rhinitis

Allergic rhinitis is one of the most common atopic conditions worldwide. It accounted for up to 20–25% of the population in Canada [2], 25% in South Korea [3] 23% in Europe [44], and 16.1% in the USA [4]. In recent years, the incidence was also high in developing countries [17]. For example, in China, allergic rhinitis accounted for 8.7 to 24.1% of the population in eleven major cities [14]. Besides, its prevalence also increases with age in adolescence [45], as evidenced by 8.5% in 6–7 years old versus 14.6% in 13–14 years old (ISAAC phase III study, which consists of data from 236 centers in 98 countries) [46]. Clinically, patients with allergic rhinitis present with congestion and inflammation of mucous membranes of the upper respiratory tract, including nasal cavity, throat, eyes, and ears [17, 33, 36]. Nasal itchiness, sneezing, and runny nose are common.

#### Pathogenesis

Allergic rhinitis is a chronic IgE-inflammatory disease of the nasal mucosal lining. Common culprits included indoor (like dust mites and pets) and outdoor allergens (like pollen) [2–4, 14, 17, 46, 47].

The nasal mucosa is innervated by a group of sensory nerve fibers including parasympathetic nerves. Sensitization to allergens starts from the nasal epithelium barrier [5, 17]. Early-phase allergic response is triggered by allergen-induced degranulation of nasal mucosal mast cells [5]. The released histamine promotes the expression of H1 (H1R) and H2 (H2R) receptors in the nasal vessels. Histamine binds H1R, a G protein-coupled receptor (GPCR) through Gq/11, activating PLCγ and stimulating IP3 and DAG production. This generates Ca^2+^ and PKC. Ca^2+^ promotes nasal vasodilation, while PKC activates NF-kB and thus enhances the proinflammatory gene transcription. These processes increase pericellular permeability and augment the antigen capture and processing ability of DCs. H2R binds to GαS protein, activating adenylyl cyclase (AC) and stimulating cyclic adenosine monophosphate (cAMP). The above increases vascular permeability and vasodilation, causing nasal mucosal congestion and watery nose. Besides, the sensory nerve stimulation induces acetylcholine release from the parasympathetic nerves. Signals are transmitted to the central nervous system, causing motor reflexes like nasal itching and sneezing.

Sustained inflammation and prolonged allergic reactions are induced by eosinophils, basophils, and Th2 cells in late-phase allergic response [19, 43]. This prolongs the symptoms of nasal congestion, sneezing, and runny nose.

### Allergic Asthma

Allergic asthma is another common atopic condition. Its incidence varies greatly with different countries: 30.1% in South Korea [3], 28.4% in Australia [48], 16.4% in the USA [4], 8–12% in Canada [2], and 8.2–9.4% in Europe [49]. With increasing air pollution, and the change of agricultural farming to home petting, the incidence of asthma is on the rise [16, 27]. Patients with allergic asthma are often presented with coughing, wheezing, shortness of breath, and chest tightness, with variable expiratory airflow limitation [29].

### Pathogenesis

Allergic asthma is also caused by IgE mast cells and eosinophils. It is a complex heterogeneous disease, characterized by chronic airway inflammation and structural changes in the lung. Similar to allergic rhinitis, it can be induced by indoor (e.g., dust mites, pets) and outdoor allergens (e.g., pollens) [2, 4, 29, 48]. Besides, it can be triggered by other factors such as exercise, viral infections, extremes of weather, and stress.

Upon allergen exposure, the airway epithelial cells are critical in providing rapid response by secreting mucus and initiating inflammation. This is important as a barrier to protect against infections from airborne pathogens. Thus, defective barrier function or viral infection could result in asthma [29].

Allergic asthma is triggered by submucosal mast cells activation in the lower airway, releasing histamine, PGD2, LT, PAF, and Th2 cytokines in early-phase allergic response [16, 25]. H1 receptor-mediated vasoconstriction and H2 receptor-mediated vasodilation results in biphasic response of pulmonary arteries by histamine. Histamine, LT, and PAF affect the bronchial microcirculatory system and cause plasma leakage from the microcapillary venules. This results in coughing, wheezing, shortness of breath, and chest tightness [21, 43]. In late-phase allergic response, elevated Th2 cytokines including IL- 5 and IL- 4 lead to increased eosinophil recruitment, while IL- 13 activates the mucus-producing gene in goblet cells, Muc5ac, and results in overproduction of mucus [50]. The release of ECP causes further damage to the epithelial barrier, on top of the insult by allergens and toxins secreted by microbes [43, 50]. Insults to the epithelial barrier can be dysregulated, resulting in the laying down of matrix structures like collagen instead of functional epithelial cells. Loss of barrier functionality makes the epithelium more defective and more susceptible to remodeling [50].

## Characterization of Inhalant Allergen Molecules

Allergens are usually harmless substances but can induce specific IgE-mediated type I hypersensitivity responses in atopic individuals [30]. Allergens can be broadly categorized as outdoor versus indoor and biological versus chemical allergens [29, 47]. Common outdoor allergens can come from biological origin such as pollens, fungi, and molds [8, 9, 48]. Indoor allergens have a huge impact on most, as people often spend more than 90% of their time indoors [24, 26]. They consist of dust mites, dogs, cats, cockroaches, and molds. Indoor allergens are also impacted by air pollutants, which depend on the amount of air penetrating from the outside and presence of indoor air pollution sources. Different sensitization patterns for inhalation allergens are summarized in Tables 2 and 3.

**Table 2:** Summary table for allergen sensitization and atopic diseases in different localities

Location/years of study	Patient demographics	Allergen sensitization	No. of allergens sensitized	Atopic disease associated (%)	Publication
Asia					
East China (Chang-zhou)/2018–2019	*N* = 1246 - Males: 65.54% - Females: 34.46% - 1–14 years: - 1–3: 6.5% - 4–7: 40.14% - 8–14: 53.36% - 80.05% with positive sIgE to inhaled allergens	Der p 1: 65.38% (HDM: 20.67%) Mold mix: 25.56% Dog hair: 13.94% Cat dander: 11.3% Amaranth: 6.41% Tree pollen mix: 94.15% Cockroach: 3.77% Mulberry: 2.64% Grass mix: 2.48%	Increased inhaled allergen sensitization with age (years): - 1–3: 61% - 4–7: 71% - 8–14: 89% - For Der p 1, HDM, cat, mold mix, amaranth	(in 11 major cities in China) 8.7–24.1% for AR - Increasing yearly	[14]
Vietnam/2014–2021	- *N* = 423 - Males: 46.8% - Females: 53.2% - 5–84 years: - Children: 18.7% - Adults: 81.3% - *Only the patients with positive SPT to* ≥ *1 allergens were included*	HDM (Der f: 59.8%; Der p: 50.4%; Bt: 49.6%; other storage mite: 10.4%) Cockroach: 10.2% Cat hair: 8.2% Fungi (*Aspergillus* species: 6.9%; *Cladosporium* species: 2.7%; *Alternaria* species: 1.3%) Bermuda grass: 2.1% Dog hair: 2.6% Mouse hair: 0.5%	94% with positive results to ≥ 1 HDM: - 31.5% positive to Df and Dp - 25.7% positive to Df and Bt - 26.7% positive to Dp and Bt - 18.2% positive to Df, Dp, and Bt - More frequently simultaneously sensitized to cockroach and cat hair	18.2% with AR	[53]
South Korea/2011–2016	- *N* = 2791 males: 38.7% - females: 61.3% - 50.9 ± 16.1 years - Group I: 45.2 ± 13.5 years (*N* = 2166) - Group II: 70.7 ± 4.6 years (*N* = 625) - 31.2% (871/2791) with positive SPT		Group I (45.2 ± 13.5 years)	< Group I vs group II > - AR: 25.0% < 28.1% vs 14.1% > - Chronic rhinosinusitis: 18.3% < 16.7% vs 24.0% > - Bronchial asthma: 30.1% < 29.1% vs 33.6% >	[3]
		Der p/Der f	21.4%/21.6%		
		Alder/birch/oak	8.4%/8.2%/5.9%		
		Cat epithelia	4.7%		
		Mugwort	3.8%		
		Dog epithelia	2.8%		
		Japanese hop	2.5%		
		Grass mix	1.7%		
		*Alternaria*	1.1%		
		Cockroach: 2.4% (1.6–2.6%) *Aspergillus*: 0.5% Ragweed: 1.4% (1.0–1.6%)			
North America					
Canada/2014	*N* = 623 - males: 36.9% - females: 63.1% - 4–84 years (mean: 38.6 years) - 70.3% (438/623) with positive SPT	Cat: 53.1% HDM: 50.3% Grass: 39.2% Birch: 23.7% *Alternaria*: 23.7% Dog: 17.3% Poplar: 12.1% Cedar: 9.6% *Aspergillus*: 9.6% *Hormodendrum*: 8% *Penicillium*: 6.2%	1: 25.1% (110/438) 2–3: 45.4% (199/438) ≥ 4: 29.5% (129/438)	12% of children and 8% of adults suffer from asthma 20–25% suffer from AR	[2]
Australia					
Australia/2007– 2008	*N* = 2226 - Males: 49.1% - Females: 50.9% - 6.1–12.6 years (mean: 10.1 years) - 44.4% with positive SPT	HDM: 36.1% Cat: 8.6% Cockroach: 7.2% Ryegrass: 17.7% Grass mix: 12.9% *Alternaria*: 9.8% *Aspergillus*: 3.4%	≥ 1 indoor allergens: 37.6% ≥ 1 outdoor allergens: 22.9%	28.4% had ever had asthma diagnosis in lifetime 15.1% with current asthma	[48]
Africa					
Africa (Angola)/2017	*N* = 1023 - males: 51.6% - females: 48.4% - 5–14 years: - 5–9: 42.4% - 10–14: 57.6% - 8.0% (82/1023) with positive SPT	HDM (Der p: 4.9%; Der f: 2.2%; Bt: 2.0%) Cockroach: 2.2% Dog epithelium: 0.4% Weed pollen mix: 0.4% Cat epithelium: 0.3% Fungi (*Alternaria alternata*: 0.3%; *Mucor mucedo*: 0.3%; *Aspergillus fumigatus*: 0.2%; *Cladosporium herbarum*: 0.2%) Grass pollen mix: 0%		1: 78.1% (64/82) ≥ 2: 21.9% (18/82) - Most frequently observed polysensitizations involved co-sensitization to mites and cockroach	[47]

*SPT* skin prick test, *HDM* house dust mite, *AR* allergic rhinitis, *Can f 1 Canis familiaris 1*, *Der f 1 Dermatophagoides farinae*, *Der p 1 Dermatophagoides pteronyssinus*, *Bt Blomia tropicalis*, *Fel d 1 Felis domesticus*

**Table 3:** Summary table for allergen sensitization and atopic diseases in different European countries [15, 44, 49]

Allergen sensitization (%) by SPT	All	AT	BE	SZ	GM	DK	FI	FR	GR	HU	IT	NL	PL	PT	UK
Grasses	33.4	20.2	24.5	71.0	34.1	64.0	18.5	19.3	42.3	37.3	18.6	34.4	30.8	31.6	50.8
Der p	26.5	12.6	29.9	22.1	17.0	40.9	15.2	29.2	26.2	26.1	34.7	29.0	16.8	65.3	31.7
Cat	19.4	11.5	18.1	24.8	23.6	32.0	26.2	14.8	15.4	22.6	17.6	18.5	16.3	15.0	27.8
Dog	16.4	8.8	15.0	13.1	18.0	29.4	24.6	14.2	10.8	20.8	13.4	28.3	12.3	7.7	15.9
Ambrosia	10.7	5.3	3.0	9.7	9.3	14.3	1.4	4.5	5.1	49.7	3.1	16.7	5.4	10.8	7.1
Blatella	5.7	2.2	1.4	0.9	6.8	10.7	3.6	8.0	7.0	0.6	3.0	8.8	6.8	21.0	0.0
	Tree pollens: - Hazel: 17.1% (3.9–37.8%); alder: 16.2% (2.3–36.2%); birch: 19.6% (4.0–49.1%); plane: 3.3% (0.0–11.9%); cypress: 2.6% (0.0–8.7%); olive: 11.5 (1.0–32.4%) Weed pollens: - *Artemisia*: 12.6% (3.2–38.8%); *Parietaria*: 7.5% (0.7–30.7%) Fungi/mold: - *Alternaria*: 6.1% (0.0–18.7%); *Cladosporium*: 3.0 (0.0–8.5%); *Aspergillus*: 2.7% (0.0–7.1%)														

(*N* = 3034, median age = 33 years); prevalence of asthma (2013): 8.2% in adults, 9.4% in children; prevalence of AR (2001): 23% (17–29%) [15, 44, 49]

*SPT* skin prick test, *Der p Dermatophagoides pteronyssinus*, *AT* Austria, *BE* Belgium, *SZ* Switzerland, *GM* Germany, *DK* Denmark, *FI* Finland, *FR* France, *GR* Greece, *HU* Hungary, *IT* Italy, *NL* The Netherlands, *PL* Poland, *PT* Portugal, *UK* United Kingdom

Climate change and air pollution play important roles in altering aeroallergen content [15, 34]. Global warming prolongs pollen seasons, increasing the frequency, duration and severity of pollen exposures [29, 31]. It favors the growth and spread of highly invasive plant species (e.g., ragweed) into areas that were never present before. Besides, it also increases the formation and spread of air pollutants like ground-level O_3_, dust storms, infections, thunderstorms, and wildfires. Indeed, wildfire smoke is an ambient natural source of many air pollutants. Air pollution and climate change can affect both the quality and quantity of allergenic protein in pollen grains [9, 27, 34] via various mechanisms. For example, pollutants in the air can induce chemical modifications in pollens and agglomerate pollens; climate change can affect the bioavailability and abundance of pollens through changes in the rainy season, vegetation cover, and pollen season [16]. In general, increased allergen abundance in the air exacerbates inhalation-triggered allergic conditions such as asthma.

Inhalant allergens are found in airborne substances that you breathe in. It can be biological or chemically derived. Here we will discuss the characteristics of common biological allergens, their epidemiology of sensitization, and pathophysiology of allergic sensitization.

### Dust Mite (DM) Allergy

DM are one of the most common indoor allergens [16, 19, 24]. High indoor humidity (at least 50%) and high temperatures (20 to 25 °C) favor the spread of DM [44]. DM are commonly found in curtains, furniture, beddings, carpets, and other soft furnishings. The dead skin cells humans and animals expel, which is common in locations with significant dust buildup, are food sources for DM. Allergenic proteins (mainly proteases) of dust mites are present in their body parts, and persistent in their feces.

The molecular identities of DM allergens are well-defined; they include *Dermatophagoides pteronyssinus* (Dp; major allergen: Der p 1, Der p 2, Der p 23) [51, 52], *Dermatophagoides farinae* (Df; major allergen: Der f 1, Der f 2) [51], and *Blomia tropicalis* (Blomia; major allergen: Blo t 1) [30], with variation in distribution according to locations. Df (59.8%) sensitization is more prevalent than Dp (50.4%) and Blomia (49.6%) in Vietnam patients with allergy [53]. Dp (85%) sensitization is similar to Df (83.5%) in Southern China [5]. Patients often have co-sensitization to more than one type of dust mites [53]. Besides, Der p 10 shares homology with tropomyosin of shrimp and crab as a cross-reactive allergen [51]. Therefore, Der p 10 monosensitization can signify food rather than dust mite allergy.

#### Epidemiology of Allergic Sensitization to Mites

DM is the dominant allergen associated with allergic rhinitis and asthma worldwide [2, 14, 15, 53]. Early exposure to high levels of dust mite allergens in the perinatal period is associated with asthma later in life [32]. In general, dust mite sensitization is more common in the parts of the world with higher humidity, e.g., East China (65.38% for Dp) [14], Canada (50.3%) [2], and Portugal (65.3%) [15]. It is less common in regions with lower humidity (36.1% in Australia [48], 21% in South Korea [3], 12.6% in Austria [15]) and tropical countries with poor hygiene (prevalence of Dp and Df sensitization in Angola, Africa, is 4.9% and 2.2%, respectively [47]).

#### Pathophysiology of Allergic Sensitization to Mites

Take Dp as an example. Der p 1 is the major allergen, while Der p 2 is another important allergen [8]. Besides the action of increasing epithelial barrier permeability, Der p 1 also cleaves lymphocyte surface receptors, like IL- 2 receptor (CD25) and FcεRII. This cysteine protease digests other proteins, as well as itself, producing fragments of altered allergenicity. Der p 2 is a homolog of the adapter protein and myeloid differentiation protein 2 (MD- 2). It facilitates lipopolysaccharide (LPS)-mediated signaling through Toll-like receptor (TLR) 4, increasing allergen update as an “autoadjuvant.”

Besides, the dust mite fecal particles also contain mite DNA, bacterial DNA, endotoxin, and chitin [16]. They can act as adjuvants by triggering innate signaling pathways. Notably, mite DNA and bacterial DNA are unmethylated and are natural TLR9 ligands. TLR9 is expressed by B cells, monocytes, NK cells, and plasmacytoid DCs. TLR9 activation leads to the production of various proinflammatory cytokines such as type I interferon, IL- 6, TNF, and IL- 12. LPS is a TLR4 ligand. TLR4 signaling triggers the MyD88 and TRIF dependent pathway. MYD88-dependent pathway induces proinflammatory cytokines production, and TRIF-dependent pathway induces the production of type I interferon and cytokines. Chitin is a C-type lectin. Interestingly, the acidic mammalian chitinase (AMCase) has been linked to asthmatic inflammation [54].

### Pet Allergy

Over 50% of US and European families have household pets, with dogs being the most common, followed by cats [16]. They act as a major source of indoor allergens, released through secretions, excretions, and dander. The prevalence of allergies to dogs has increased because of increasing ownership. Allergens are contained in their hair, shed skin cells, saliva, and urine.

#### Epidemiology of Allergic Sensitization to Pets

Dog sensitization varies on their prevalence in households and mirrors that of cat sensitization. The sensitization rate is higher in Denmark (29.4%) and Netherlands (28.3%) [15], but much lower in China (13.94%) [5], Vietnam (2.6%) [53], South Korea (0–2.8%) [3], and Portugal (7.7%) [15]. In general, they are considered less allergenic and less frequently blamed for allergic reactions [16]. To date, six dog allergens have been identified (Can f 1–6) [55]. Can f 1 is a salivary lipocalin protein [56] and can be easily transferred to fur and skin when dogs groom themselves or shed to the environment, which then easily remains airborne for prolonged periods. Sensitization to dog allergens is associated with asthma and allergic rhinitis [8, 16].

Cats are another common allergen source resulting in allergic rhinitis and asthma. The incidence of its sensitization is higher in industrialized nations like Canada (53.1%) [2], Denmark (32.0%) [15], and the UK (27.8%) [15]. On the other hand, its sensitization is lower in China (11.3%) [14], Vietnam (8.2%) [53], South Korea (0.8–4.7%) [3], and Australia (8.6%) [48].

**Fig. 2:**
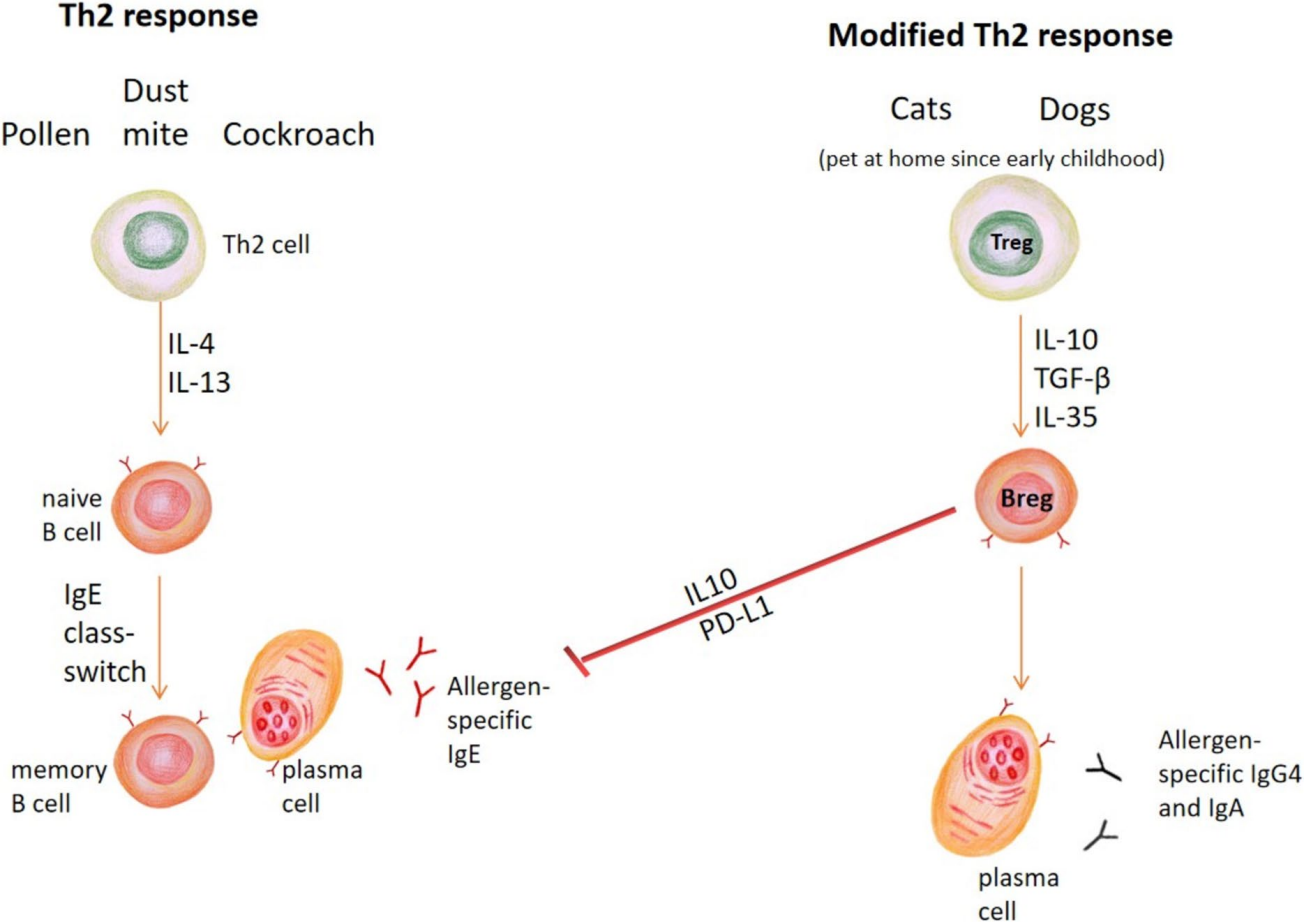
Schematic representation of Th2 versus modified Th2 immune response. Sensitization by pollens, dust mites, and cockroaches induce classic Th2 response, resulting in IL- 4 and IL- 13 secretion, which stimulate IgE class-switch in B cells and plasma cells to produce allergen-specific IgE (sIgE). On the other hand, intense sensitization by cats and dogs can induce “modified Th2 response” if they are kept as pets at home since early childhood. Induced regulatory T (Treg) cells secrete IL- 10, TGF-β and IL- 35, which induce regulatory B (Breg) cell formation. This inhibits allergen sIgE secreted by B cells and plasma cells. The allergen-specific IgG4 and IgA antibodies produced by Breg also exert blocking actions. This induces “immune tolerance.”

Nevertheless, there are several studies demonstrating the paradoxical effect of intensive cat exposure in early childhood, especially in the first year of life [26]. This potentially results in a modified Th2 response (Fig. 2), associated with high IgG4 to cat allergens, especially to Fel d 1 and lower risk of cat allergy [57]. Interestingly, in countries with higher exposure to pollen, dust mite, and cat allergens, there is less cat sensitization than that for mite or grass pollen [15, 16].

#### Pathophysiology of Allergic Sensitization to Pets

A Swedish study reported that Can f 1 (43%) and Can f 5 (33%) sensitization were the most common allergens for dog dander sensitization [58]. Moreover, polysensitization with elevated IgE levels to Can f 3 (albumin), Can f 4, and Can f 6 was more common in patients with asthma, while monosensitization to Can f 5 was more common in allergic rhinitis without asthma [58].

As discussed above, the major cat allergen, Fel d 1, can remain airborne for long periods due to its small size [16, 26]. sIgE to Fel d 1 correlates strongly with cat allergic asthma [59]. Besides, there are two other important allergens [16]: Fel d 2 (albumin) and Fel d 5w (cat IgA). sIgE to Fel d 2 cross-reacts with dog, beef, and pork albumin, resulting in pork/cat syndrome. sIgE to Fel d 5w is mostly direct against the oligosaccharide galactose *α*− 1,3-galactose (α-gal). The latter is induced by tick bites and can result in delayed anaphylaxis to red meat.

### Pollen Allergy

Pollen seasons vary geographically and seasonally [60–62]. A recent study showed that pollen allergy in spring can account for 25,000 to 50,000 asthma-related emergency department visits annually in the USA [28, 30, 34, 63, 64]. Tree pollens (like oak, birch, cedar, and pine) are released in springtime. Grass species (like Kentucky bluegrass, Bermuda grass, Timothy grass) release pollen in the late spring and summer. Weeds (e.g., ragweed, sagebrush, pigweed, lamb’s quarters) produce pollen in the late summer and autumn. Besides, the increased use of ornamental plants in parks, workplaces and homes provides new sources of aeroallergens [16]. There is extensive cross-reactivity within both grass and weeds. Pollen sensitization results in seasonal allergies like rhinoconjunctivitis, allergic rhinitis, and asthma.

#### Epidemiology of Allergic Sensitization to Pollens

Pollen sensitization depends on the pollen growth of a region. Grass pollen allergy (median prevalence of 33.4%) is the top respiratory allergy in most European countries [15], reaching 71.0% in Switzerland, 64.0% in Denmark, and 50.8% in the UK. However, in regions where the pollen growth is poor, the prevalence of sensitization can be very low: 0.5–1.7% in South Korea [3] and 2.48% in East China [14]. Even in different parts of the same country with variable vegetation patterns or in different seasons, the pattern of sensitization can be different [5, 28, 65].

Ragweed pollen is one of the most common environmental allergens in the USA. A total of 15.5% of Americans are sensitive to Ragweed [66]. Ragweed pollen usually peaks in late summer and early fall. Ragweed pollen can be carried for long distances by wind. Warmer summer temperatures and increased carbon dioxide can also lead to longer pollen seasons and thus greater pollen loads [66].

On the contrary, tree pollen sensitization is more common in East China (94.15%) [14]. There is also a variable sensitization pattern in Europe depending on type of plantations and risk factors linked to westernization such as hygiene, nutrition, and pollution [15].

#### Pathophysiology of Allergic Sensitization to Pollens

Allergic asthma is manifested as inflammation of the airway driven by Th2 cytokines and IgE. It is immunologically characterized by the infiltration and activation of eosinophils, mast cells, neutrophils, and immune lymphoid cells in the airway. Asthmatic patients suffer from airway smooth muscle hyperresponsiveness, airway constriction and airway thickening, mucus hypersecretion and impaired clearance, subepithelial cells inflammation, and fibrosis [67].

Thunderstorm asthma can be triggered by rainfall, dew, fog, and watering of lawns, which can rupture pollen grains within the anthers. Disturbances like wind, recreational activities, and lawn-mowing result in the release of tiny allergenic fragments that concentrate at ground level causing severe asthma crises in people with pollen allergic asthma [68].

### Cockroaches and Molds

Cockroaches can be found in inner-city environments, especially in floor dust, kitchen cabinets, and basements. The major cockroach allergens include Per a 1 in American cockroaches and Bla g 1 and Bla g 2 in German cockroaches [16]. Patients allergic to cockroach tropomyosin (Bla g 7 and Per a 7) can be reactive to tropomyosin in dust mites and shrimps [69]. Cockroach sensitization is associated with allergic rhinitis, allergic rhinoconjunctivitis, and asthma [16]. Interestingly, atopic individuals often have co-sensitization of cockroaches with other allergens like in dust mites [47, 70] and cats [53]. The prevalence of cockroach allergy accounts for 21% in Portugal [15], 27% in China [70], and 17–41% in the USA [71]. Suggested pathophysiological mechanisms for cockroach allergens include the involvement of protease-activated receptor (PAR)− 2 for their penetration through epithelial cells and the induction of Th2 cytokines [69, 71].

Dark, humid, and poorly ventilated areas are the important sources of mold or fungi such as *Alternaria*, *Cladosporium*, *Penicillium*, and *Aspergillus* [30, 72]. Sensitization of mold or fungus is associated with allergic rhinoconjunctivitis and asthma. In general, their sensitization is low (< 10%) across different localities [3, 14, 15, 48, 53]. *Alternaria* is the most important fungal species for sensitization, as it has a universal distribution with the ability to withstand a wide range of climate conditions especially warm climate conditions [73]. Alt a 1, that is in the spore cell wall, is regarded as the predominant *Alternaria* allergen.

### Th2 Versus Modified Th2 Response

Allergens like pollens, dust mites, and cockroaches induce classic Th2 response [26, 34]. Through the process of allergen sensitization and secretion of IL- 4 and IL- 13, sensitized Th2 cells stimulate IgE class-switch in B cells and plasma cells to produce allergen-specific IgE (sIgE) (Fig. 2). The prevalence and titer of sIgE increase with exposure. For example, Der p 1, one of the major house dust mite allergens, is an active cysteine protease that can disrupt tight junctions in epithelial cells, thereby increasing allergen uptake by antigen-presenting cells [8, 12, 17]. This prevents tolerance development even at high dose.

On the contrary, allergens from cats and dogs are known to induce modified Th2 response if they are kept as pets at home since early childhood [26, 57]. Animal dander is very small and can remain in the air, stay on clothes, and be kept in carpets or furniture for a long period of time. The prevalence and risk of allergic asthma are much reduced with high exposure to these allergens during childhood. This phenomenon is associated with reduced IgE and high IgG- and IgG4-specific responses. Mechanistically, upon allergen sensitization, induced Treg cells secrete IL- 10, TGF-β, and IL- 35, which induced regulatory B (Breg) cell formation [13, 20, 24]. The production of IL- 10 by Breg cells inhibits allergen sIgE secreted by B cells and plasma cells. The allergen-specific IgG4 and IgA antibodies (up to 20%) produced by Breg also have a blocking action. This induces “immune tolerance” upon high dose exposure since early childhood. For example, Fel d 1, the major cat allergen, unlike Der p 1, is not an enzyme but shares sequence homology with Clara Cell secretory protein (CCSP), which is a uteroglobin of the bronchial epithelial cells [59]. Fel d 1 selectively induces IL- 10 as well. With cat dander being 2 to 20 µm in diameter (compared to 20 to 40 µm for dust mite), it can remain airborne for a longer period of time, and the exposure can be up to 1 µg/day (compared to ≤ 10 ng/day in dust mite) [26]. Additionally, the dander particle can easily stick to human clothing to transfer to home, school, or other buildings with no cats. IL- 10 induction and a higher regular dose of exposure facilitate immune tolerance induction.

## Chemical Contribution to the Development of Allergic Diseases

Air pollutants like diesel exhaust particles (DEP) act as an adjuvant in stimulating allergic response. Exposure to tobacco smoke and air pollution (CO, NO_2_, PM_2.5_, PAH) can result in oxidative stress, and thus significant epigenetic changes by altering histone modification (acetylation, methylation) and chromatin remodeling of proinflammatory genes [8, 32]. This possibly alters T cell balance to Th2 cells, impairs Treg function, and increases eosinophil recruitment [7, 43, 48].

Besides, volatile chemicals like fragrances can also be causative agents for asthma. There are divided views on psychological and physiological contributions [74–76]. These chemicals can result in trigeminal stimulation, and thus the release of neuropeptide mediators like substance P and calcitonin-gene-related peptides. These neuropeptides can affect respiration and airway glandular secretions, triggering asthma. Fragrance allergy can increase Th2 production of IL- 4, IL- 5, and IL- 13 [77]. Similarly, occupational allergens are causative agents for allergic rhinitis and asthma [78]. They are further classified as low molecular weight (LMW) and high molecular weight (HMW) allergens. Protein allergens are often HMW and can act as complete antigens, whereas chemical agents are LMW; they react with proteins to form a hapten-complex to functioning allergens. Examples include wheat and latex.

### Particulate Matter (PM)

Besides outdoor sources like mineral dust, vehicle exhaust, and heating combustion, PM can originate from indoor sources: tobacco smoke, incense burning, clothing residues and fiber breakage, cooking, etc. [7, 16].

PM consists of a mixture of solid and liquid particles suspended in air and is divided into different categories based on the aerodynamic diameters: coarse PM_10_ (≥ 10 µm), fine PM_2.5_ (≤ 2.5 µm), and ultrafine PM_0.1_ (< 0.1 µm) [16]. The particle size influences the PM’s capability to penetrate deeply in the lung. PM_10_ can penetrate the upper respiratory tract (nose, throat, larynx), PM_2.5_ can penetrate into the tracheobronchial tract (trachea, bronchiole), and PM_0.1_ can penetrate into the alveolar region.

PM_2.5_ acts synergistically with allergens by increasing allergenicity and bioavailability [8, 16]. Besides causing dose-dependent disruption of the pulmonary endothelial cell barrier (Fig. 1), PM induces the generation of truncated products of phospholipid oxidation, destabilizing cell junctions. The increased reactive oxygen species (ROS) production leads to direct oxidative damage. Besides, by activating redox-sensitive transcription factor NFkB, ROS induces the production of pro-inflammatory IL- 6, IL- 8, and IL- 1β and thus further disrupts barrier function. PM also promotes the production of epithelial cytokines, like IL- 33 and TSLP, which induce Th2 cytokine (i.e., IL- 4, IL- 5, IL- 13) synthesis [27]. The above generates a positive feedback loop of increased epithelial permeability. This results in airway irritation and inflammation.

## Diagnostic Tests and Management of Aeroallergen Sensitization

Skin test and blood test are used in the clinical diagnosis of allergen sensitization [8, 11–13, 17, 18, 20, 43, 79, 80]. Here, we briefly describe skin prick test, skin patch test, sIgE assay, and basophil activation test.

### Skin Test

Skin prick tests (SPTs) [12] are widely used as they are safe, fast, simple, and cost-effective. Their response relies on an intact immune system and the presence of IgE-sensitized active mast cells that release mediators [81]. In skin tests, the skin is exposed to suspected allergens and then checked for signs of allergic reactions. A wheal and flare response caused by histamine release at 15 min confirms IgE sensitization and IgE activation of skin mast cells in response to the allergen extract used. With a high negative predictive value, SPTs help with immediate patient management in the clinical settings.

However, we should caution that controlled amounts of standardized allergenic extracts are preferred, and allergenic proteins for testing should be quantified to ensure reliability and reproducibility of SPT results [81]. In case of improper storage conditions, protein extracts can lose their allergenicity as protein is acted upon by proteolytic enzymes [81]. Besides, it would be difficult for patients with dermatographism, extensive skin lesions like eczema, or those who cannot be put off antihistamines [12, 81]. Cross-reactivity between different allergens due to panallergens, which is quite common between different pollen allergens, also makes the interpretation difficult [51].

Skin patch tests can be applied for chemical allergies, as they are capable of causing skin sensitization [76, 77]. They can be either manually loaded or preloaded commercially. Nevertheless, the test is mainly for contact allergy diagnosis and can be affected by the preparations from different manufacturers, using different patch test systems and non-standardized patch testing procedure [76]. In the case of using petroleum-based patch test preparation, advance preparation of fragrance test material can result in allergen evaporation and false negative results [76, 77]. Similar to SPT, poor skin condition makes the test impossible.

### Allergen-Specific IgE (sIgE)

Testing for allergen-specific IgE (sIgE) [12, 13] is convenient, standardized, and suitable for patients with skin test limitations (e.g., poor skin conditions, use of antihistamines before skin tests). However, a positive test can indicate sensitization rather than a genuine allergy. In the case of a very high total IgE, there can be non-specific sIgE results [12, 81]. In addition, its applications can be limited by its high cost, limited specific allergen panels for testing, and lack of standardized quantifications between different commercial assays. More sensitive and specific tests are needed for sensitization to two or more related allergens.

The detection of sIgE can be performed with either whole allergenic extracts or individual allergenic molecules/epitopes (also known as component-resolved diagnostics (CRD)) [12, 13]. CRD has its advantages as it can differentiate genuine primary sensitization from cross-reactivity due to the presence of IgE towards panallergens [12, 13, 82]. In a study on dog dander sensitization on 313 Swedish adults [58], Can f 1 (43%) and Can f 5 (33%) sensitization were the most common. It is worth mentioning that allergens can be under-represented in SPT solutions, e.g., Can f 6 and Can f 2 are in very low amounts while Can f 3 is highly variable (9–98%) [83]. There are also cases that have positive sIgE to the whole extract but are negative to CRD; it can be due to presence of α-gal in the whole extract and thus not genuine sensitization. Among 348 children and 467 adults with seasonal allergic rhinitis in nine Southern European cities [64], grass pollen (Phl p 1 and/or Phl p 5) was most sensitized yet with broad variability among different localities. In several Colombian cities [84], sensitization to Blo t 21 and Blo t 5, but not Blo t 2, was associated with asthma. In South China [52], Der p 1, Der p 2, Der p23, Der f 1, and Der f2 are the primary sensitizing components in mite-sensitized respiratory allergy. However, Der p 10 monosensitization means tropomyosin allergy, and food allergy should be investigated instead of going for aeroallergen immunotherapy [13, 51]. Clearly, knowledge about genuine sensitization versus cross-reactivity towards panallergens will help in better decision on allergen immunotherapy (AIT).

### Basophil Activation Test (BAT)

As discussed in previous sections, the cross-linking of FcεRI-bound IgE on mast cells and basophils by allergens lead to their activation and degranulation. Therefore, basophil activation can be used as a surrogate marker for mast cell reactivity and is applied in the basophil activation test (BAT) [12, 85]. With the stimulation by allergens (can be in different dilution series), positive controls (examples include monoclonal antibodies against FcεRI and bacterial polypeptide *N*-formylmethionine-leucyl-phenylalanine/fMLP), and negative control, activated basophil can be detected by CCR3, and CD63 or CD203c by flow cytometry [86]. BAT can be used as a biological assay for aeroallergens (like dust mites and pollen), food or drug allergy, and monitoring AIT [12, 86]. It is considered a functional assay, with high specificity (up to 100% in Df [86]).

However, the analysis of BAT should be cautioned because of the variable sensitivity with different allergens, the requirement of technical expertise, and the timely requirement in processing of fresh blood samples. In case of lack of circulating sIgE antibodies such as in local allergic rhinitis, FcεRI-bound IgE might not be present on circulating basophils and can lead to a false negative result [86]. Moreover, the allergen concentrations and cut-offs of BAT are not universally standardized [12, 86]. In addition, there can be patients with unresponsive basophils (as reflected by lack of basophil response to positive controls) which render the test impossible.

### Insight Into Management of Allergic Diseases

Despite our knowledge on the pattern of allergen sensitization (via SPT, sIgE, and BAT), avoidance is still the simplest way in preventing inhaled allergy symptoms [8, 16, 79].

Regulatory T (Treg) cells and regulatory B (Breg) cells play important roles in the pathophysiology of aeroallergen sensitization and respiratory allergic diseases (Fig. 1) and are employed in allergen immunotherapy (AIT) [8, 12, 17, 18, 20, 43, 73]. Treg cell is a subset of CD4 + T cells characterized by a high level of α-subunit IL- 2 receptor (CD25) expression, prominent expression of the transcription factor forkhead box 3 (FOXP3), and lack of IL- 7 receptor α-subunit (CD127) expression [18, 87]. During AIT, a high-dose allergen exposure induces regulatory dendritic cell (DC) markers like complement component 1 and stabilin- 1, stimulating their secretion of IL- 12, IL- 27, and IL- 10 and attenuating CD86 expression [12]. IL- 12 activates STAT4, which drives IFNγ gene expression and secretion, promoting the differentiation of Th1, and suppresses IL4 gene locus and subsequently Th2 differentiation [12]. IL- 10, in the presence of TGF-β, drives naive T cell proliferation into allergic-specific Treg cells [18]. Treg cells express CTLA- 4 and cell-bound TGF-β and secrete regulatory cytokines like IL- 10, TGF-β, and IL- 35 [12, 13]. Binding CTLA- 4 to co-stimulatory molecules CD80/CD86 on DCs reduces their expression. IL- 10, TGF-β, and IL- 35 upregulate FoxP3 and suppress GATA3 expression, thus promoting Treg cell differentiation and suppressing Th2 [11, 13, 87]. This results in shifting towards a Th1 response. Less eosinophils are activated [43]. These cytokines also suppress the activation and proliferation of effector T and B cells [13, 43].

Breg cells are a distinct sub-population of B cells whose central role is to suppress the immune ongoing immune responses and maintain immune tolerance and homeostasis. Breg cells are characterized by the production of IL- 10, IL- 35, and TGF-β, as well as the presence of cell surface proteins like CD1 d and PD-L1. IL- 10, TGF-β, and IL- 35 secreted by Treg during AIT induce Breg formation. Breg produces IL- 10 and expresses PD-L1, inhibiting the production of IgE produced by B cells and plasma cells [11, 20, 88]. Besides, Breg cells also produce blocking allergen-specific IgG, IgG4, and IgA antibodies. Serum IgG4 competes with IgE for allergen binding, inhibiting the formation of allergen-IgE complexes and reducing their cross-linking of FcεRI on mast cells and basophils [13, 17, 89]. This prevents their degranulation and histamine release. Inhibition of cross-link of low affinity IgE receptor (FcεRII, CD23) on B cells reduces IgE-facilitated allergen presentation to T cells. This reduces IgE-driven Th2 responses. Overall, inflammatory allergic response is regulated.

IL- 10-producing regulatory ILC2 is recently recognized as another player in the AIT [90]. They are identified by CD127, KLRG1, and CRTH2 surface markers. During AIT, they are induced by IL- 7, IL- 33, and retinoic acid. Besides the production of IL- 10, regulatory ILC2 also regulates allergic reactions by preventing epithelial damage via reducing the production and release of pro-inflammatory epithelial mediators, IL- 6 and IL- 8. Th2 cell responses are also attenuated [90].

To target different cytokines in aeroallergen sensitization and respiratory allergic diseases, biologics are developed for monotherapy or supporting AIT [13, 19]. Omalizumab, which targets IgE, is indicated for moderate to severe persistent asthma inadequately controlled with inhaled corticosteroid [19] and to support AIT in asthma patients [13]. There are several biologics targeting Th2-associated cytokines and their receptors: dupilumab (anti-IL- 4 and IL- 13 signaling), mepolizumab and reslizumab (anti-IL- 5), benralizumab (anti-IL- 5 signaling) [19]. They are used as an add-on maintenance therapy for severe asthma with eosinophilic phenotype. Dupilumab also improves subcutaneous AIT tolerability in AR triggered by grass pollen [13]. Tezepelumab, which targets TSLP, is indicated for add-on maintenance therapy for inadequately controlled severe asthma and enhances AIT efficacy in AR triggered by cat allergy [13, 19].

## Conclusion and Perspectives

The rising number of respiratory diseases caused by inhalant allergens poses significant challenges to health care. Although joint ventures of clinical and wet bench research have greatly advanced our knowledge on the immunological, pathophysiology and molecular mechanisms of inhalant mediated allergic diseases such as asthma and allergic rhinitis, inhalant allergen sensitization differs from region to region. In addition, the impact from epidemiological studies is much hampered by study design and cohort variations. For example, some regions like Africa have low rates of allergy and aeroallergen sensitization due to various reasons such as poor hygiene conditions, sub-par standard of health care, and hence under-representation. In many countries with low allergist-to-population ratio, family physicians carry the load of “allergist.” With the important immunological roles of both IgE and basophils, SPT, sIgE, and BAT to aeroallergen are valuable diagnostic tools. Through the understanding of the pathophysiology of respiratory allergic diseases including cytokines and roles of different immune cells, AIT and biologics are becoming more eminent in the clinical care of inhalant allergies. Building upon this foundation and growing knowledge on the underlying immunological and molecular mechanisms of inhalant mediated allergic responses, regimen directed at designing “modified Th2 response” and allergen-specific vaccines for allergic respiratory disorders will be highly desired by both the patients and the health care community.

## Data Availability

No datasets were generated or analysed during the current study.

## Abbreviations

AIT: Allergen immunotherapy
α-gal: *α*- 1,3-Galactose
BAT: Basophil activation test
Breg: Regulatory B cell
DAG: Diacylglycerol
DCs: Dendritic cells
DM: Dust mites
Df: *Dermatophagoides farinae*
Dp: *Dermatophagoides pteronyssinus*
ECP: Eosinophil cationic protein
FcεRI: High-affinity receptors
GATA3: GATA binding protein 3
H1R and H2R: H1/H2 receptors
ILC2 s: Innate lymphoid cells
IP3: Inositol triphosphate
ITAMs: Immunoreceptor tyrosine–based activation motifs
JAK: Janus-family tyrosine kinase
LPS: Lipopolysaccharide
LT: Leukotriene
MBP: Major basic protein
PAF: Platelet-activating factors
PAH: Polycyclic aromatic hydrocarbons
PGD2: Prostaglandin D2
PKC: Protein kinase C
PLCγ: Phospholipase Cγ
PM: Particulate matter
PM_2.5_: Fine particulate matter
ROS: Reactive oxygen species
sIgE: Specific IgE STAT6: transcription- 6
Tfh: T follicular helper
TGF: Transforming growth factor
TLR: Toll-like receptor
TNFs: Tumor necrosis factors
Treg: Regulatory T cells
TSLP: Thymic stromal lymphopoietin
